# A vesicular stomatitis virus-based African swine fever vaccine prototype effectively induced robust immune responses in mice following a single-dose immunization

**DOI:** 10.3389/fmicb.2023.1310333

**Published:** 2024-01-05

**Authors:** Yunyun Ma, Junjun Shao, Wei Liu, Shandian Gao, Decai Peng, Chun Miao, Sicheng Yang, Zhuo Hou, Guangqing Zhou, Xuefeng Qi, Huiyun Chang

**Affiliations:** ^1^State Key Laboratory for Animal Disease Control and Prevention, Lanzhou Veterinary Research Institute, Chinese Academy of Agricultural Sciences, Lanzhou, Gansu, China; ^2^College of Veterinary Medicine Northwest A&F University, Yangling, Shanxi, China

**Keywords:** African swine fever virus, vaccine prototypes, vesicular stomatitis virus, safety, immune potency

## Abstract

**Introduction:**

African swine fever (ASF) is a highly contagious hemorrhagic fever disease in pigs caused by African swine fever virus (ASFV). It is very difficult to control and prevent ASF outbreaks due to the absence of safe and effective vaccines.

**Methods:**

In order to develop a safe and effective ASF vaccine for the control and prevention of ASF, two ASFV recombinant vesicular stomatitis virus (VSV) live vector vaccine prototypes, containing the gene of p72, and a chimera of p30 and p54, were developed based on the replication-competent VSV, and named VSV-p72 and VSV-p35. The immune potency of VSV-p72 or VSV-p35 alone and in combination was evaluated in BALB/c mice via intramuscular and intranasal vaccination.

**Results:**

The results indicated that whether administered alone or in combination, the two vaccine prototypes showed acceptable safety in mice and, more importantly, induced high-level specific antibodies against p72, p30, and p54 of ASFV and a strong cellular immune response 28 days after vaccination. The sera from mice vaccinated with the vaccine prototypes significantly inhibited ASFV from infecting porcine alveolar macrophages (PAMs) *in vitro*. Most notably, the immunized sera from a mixture of VSV-p35 and VSV-p72 inhibited ASFV from infecting PAMs, with an inhibition rate of up to 78.58%.

**Conclusion:**

Overall, our findings suggest that ASFV recombinant VSV live vector vaccine prototypes may become a promising candidate vaccine for the control and prevention of ASF.

## 1 Introduction

African swine fever (ASF) is one of the most virulent viral diseases and is characterized by fever, hemorrhage, and high mortality. An outbreak of ASF imposes devastating economic losses on the pig industry. In 1921, the disease initially emerged in Kenya, subsequently spreading to the Caucasus region of Georgia in 2007, and gradually disseminating to neighboring countries (i.e., Armenia, Azerbaijan, Russia, and Belarus), seriously affecting the global pig-farming industry (Rowlands et al., 2008; Costard et al., 2009). In recent years, thousands of ASF outbreaks have been reported in Asian countries. The first case of ASF was confirmed in China in 2018, after which the disease broke out and spread across the country, causing huge economic losses due to the absence of effective therapeutic drugs or vaccines (Gallardo et al., 2014). Therefore, it is imperative to develop effective and safe vaccines against African swine fever virus (ASFV) for controlling and preventing ASF.

ASFV, as the pathogen of ASF, is the only member of the *Asfarviridae* family and the only known DNA arbovirus (Tulman et al., 2009; Gaudreault et al., 2020). The virion genome length is 170–190 kb and encodes more than 160 distinct proteins. Currently, there are approximately 30 kinds of proteins that have been identified as immunogenic, including p30, p54, p72, CD2v, pB475L, pC129R, pE199L, pE184L, and pK145R. Of these, p30, p54, and p72 proteins are important structural proteins and dominant antigens, and their immunological and biological functions have been intensively researched, including in virus replication, packaging, and other processes (Xu et al., 2023). Previous studies have confirmed that the p30 protein is a crucial protective antigen and inhibits ASFV internalization (Gómez-Puertas et al., 1996; Neilan et al., 2004). The p72 protein is a major component of ASFV nucleocapsid and blocks ASFV attachment to the cellular surface, thereby preventing viral invasion of cells and interfering with viral replication. Moreover, the p72 protein contains conformation-neutralizing epitopes and T cell epitopes, which can trigger a strong humoral and cellular immune response (Liu et al., 2019; Wang et al., 2019). In addition, the p54 protein can block the ASFV adsorption process on the surface of host cells, which has resulted in the p54 protein being used for the development of the ASFV vaccine as a potent immunogen (Zhao et al., 2023). The immunization with either p30 and p54 or a chimeric protein of p30 and p54 can elicit the production of neutralizing antibodies in pigs, leading to significant modification of the disease course and providing varying degrees of protection (Gómez-Puertas et al., 1998; Barderas et al., 2001). Moreover, p54 and p30 proteins that encode ASFV hemagglutinin and ubiquitin determinants were successfully fused to elicit both humoral and cellular immune responses and provided partial protection against the lethal ASFV-E75 challenge in swine (Argilaguet et al., 2012). Therefore, ASFV vaccines are widely developed based on these dominant antigen genes.

To date, the development of vaccines against ASFV has been the focus of extensive research endeavors (Liu et al., 2023; Sereda et al., 2023; Zajac et al., 2023). Conventional ASF-inactivated vaccine alone or combined with new adjuvants can induce specific antibodies in the host, but it does not confer protection against ASFV infection in pigs (Stone and Hess, 1967; Blome et al., 2014). Although natural attenuated live vaccines and gene-deficient vaccines provided efficient immune protection against homologous strains, the progress of its development has been impeded due to the violent side effects of vaccination and the possibility of virulence recovery (King et al., 2011; Abrams et al., 2013; Mulumba-Mfumu et al., 2016). In contrast, subunit and DNA vaccines provide a safer strategy with fewer side effects. Some immunogenic ASFV proteins are the main targets of the subunit and DNA vaccine, including p72, p30, p54, and p22. Previous research findings have demonstrated that immunization with ASFV p72, p54, and p30 proteins in pigs effectively induced the production of neutralizing antibodies and delayed the onset of the disease but provided little protection against virulent strains (Gómez-Puertas et al., 1996; Neilan et al., 2004; Argilaguet et al., 2011, 2012; Arias et al., 2017; Gaudreault and Richt, 2019). Jancovich et al. confirmed that the viral genome levels were significantly reduced in pig blood and lymph tissues that were immunized with 47 ASFV proteins, which indicated that immunizing animals with antigens alone did not provide protection against ASFV (Jancovich et al., 2018). Furthermore, some researchers demonstrated that pigs immunized with ASFV recombinant proteins with MONTANIDE™1313 VG N adjuvant elicited ideal humoral, mucosal, and cellular immune responses (Xu et al., 2023). DNA vaccines encoding three ASFV antigens (p54, p30, and the hemagglutinin extracellular domain) fused to ubiquitin can provide a 60% protection rate against the lethal virulent E75 strain, and there was no viremia or viral excretion in surviving pigs (Lacasta et al., 2014). These results indicated the presence of additional protective determinants in the ASFV genome. Therefore, further investigation is warranted to enhance the immunogenicity of subunit vaccines by exploring the architecture and functionality of ASFV antigen genes, as well as antigen presentation and combination.

Many studies have shown that developing safe and effective vaccines using viruses as vectors for presenting dominant antigens has become a feasible option. Previous studies demonstrated that poxvirus and adenovirus vector vaccines expressing p30, p54, p72, and pp62, or A151R, E119L, B602L, EP402R, B438L, K205R, and A104R genes from ASFV can elicit robust humoral and cellular immune responses in domestic pigs (Lokhandwala et al., 2017, 2019). Another study showed that MVA-vectored ASFV subunit antigens can induce ASFV-specific antibodies and T cell responses in swine (Lopera-Madrid et al., 2017). A Semliki Forest virus vector expressing ASFV p32 and p54 elicited robust humoral and cellular immune responses (Fang et al., 2022). Goatley et al. reported that domestic pigs immunized with pools of eight viral-vectored ASFV genes protected up to 100% of pigs from fatal disease after being challenged with virulent ASFV (Goatley et al., 2020). These results highlighted the possibility of developing ASF vaccines using a rational viral vector.

Vesicular stomatitis virus (VSV) is a single-stranded, negative-stranded, non-segmental RNA virus that primarily infects rodents, swine, cattle, and horses. The disease is characterized by vesiculation and ulceration of the oral cavity, feet, and teats, with a high transmission rate but a low clinical incidence rate, which is usually harmless (Lichty et al., 2004). Variations in the pathogenicity of different viral strains have been observed, with emerging epidemic strains reported to infect pigs and suppress innate immunity while enhancing their virulence (Velazquez-Salinas et al., 2018). Multiple preclinical studies and clinical trials revealed that genetically modified recombinant VSV vectors improved the safety of vectors and enhanced the immunogenicity of target antigens, making the development of highly pathogenic pathogen vaccines easier to achieve (Schlereth et al., 2000; Ezelle et al., 2002; Garbutt et al., 2004; Jones et al., 2005). Previous studies have demonstrated that the pathogenicity and neurotoxicity were directly related to the G protein of VSV, and the G protein of VSV New Jersey is more pathogenic than VSV Indiana in pigs (Robain et al., 1986; Martinez et al., 2003). The VSV M protein can inhibit the production of interferon and shut down protein synthesis in the host, which serves as a pivotal determinant of viral virulence. The absence of the 51st amino acid or mutations M51R, V221F, and S226R may attenuate the virulence of the virus strain (Hoffmann et al., 2010). Researchers also revealed that the safety of VSV Indiana with three amino acid mutations, M51R, V221F, and S226R, was significantly higher than that of M51R single amino acid mutant strains in swine (Fang et al., 2015). Therefore, we selected the VSV Indiana with three amino acid mutations (M51R, V221F, and S226R) to conduct research on live vector vaccines.

In this study, the VSV-based ASF vaccine prototypes were developed, and immune potency was assessed in BALB/c mice. The results revealed that vaccine prototypes were safe and elicited robust humoral and cellular immune responses after a single intramuscular or intranasal immunization in BALB/c mice, which indicated VSV is a valuable viral vector that provides a novel strategy for antigen presentation systems in the development of viral vector ASF vaccines.

## 2 Ethics statement

The rescue of recombinant virus was performed under biosafety level 2 (BSL2) conditions. All animal experiments were strictly conducted according to the guidance of the Animal Care and Use Committee of Lanzhou Veterinary Research Institute (LVRI), Chinese Academy of Agricultural Sciences.

## 3 Materials and methods

### 3.1 Cells, viruses, plasmids, mice, and serum samples

Baby hamster kidney (BHK-21) cells and human embryonic kidney 293 T (HEK293T) cells were cultured with Dulbecco’s Modified Eagle Medium (DMEM) (Gibco, Carlsbad, CA) supplemented with 10% fetal bovine sera (FBS, Thermo Fisher Scientific), 100 U/mL penicillin (Thermo Fisher Scientific), and 100 μg/mL streptomycin solution (Thermo Fisher Scientific) under a 5% CO_2_ atmosphere at 37°C. The primary porcine alveolar macrophages (PAMs) were isolated from the lung tissues of 2-month-old healthy pigs and cultured in RPMI 1640 medium (Gibco, Carlsbad, CA), supplemented with 15% FBS at 37°C with 5% CO_2_. The ASFV China/Sichuan/2019 (ASFV/CN/SC/19) was provided by the Regional Laboratory of African Swine Fever, Lanzhou Veterinary Research Institute (Lanzhou, China). The VSV-p72 and VSV-p35 strains were constructed by inserting attenuated mutations into the M gene of a recombinant VSV Indiana subtype backbone (Lichty et al., 2004). VSV-rwt was used as a control vaccine during the experiment, and the pVSV-XN2 vector sequence was synthesised and mutations (M51R, V221F, and S226R) were introduced as described previously (Hoffmann et al., 2010; Fang et al., 2015). The specific pathogen-free female BALB/c mice were purchased from the experimental animal center of LVRI. The spleen lymphocytes were isolated from the immunized BALB/c mice, then were treated with ACK buffer (Lonza, Basel, Switzerland) to lyse erythrocytes, after which they were washed with phosphate-buffered saline (PBS) twice and cultured in RPMI 1640 medium supplemented with 10% FBS subsequently. ASFV standard positive serum and negative serum were purchased from the China Institute of Veterinary Drug Control (Beijing, China).

### 3.2 The construction and rescue of VSV-p35 and VSV-p72

To construct the pVSVXN2-GFP vector, green fluorescence protein (GFP) was added between the G and L genes of the VSV genome, and then the restriction enzyme sites *XhoI* and *NheI* were imported into both ends of the GFP gene, respectively. A chimera of p30 and p54 was constructed by sequence tandem with a flexible linker, named the p35 and p72 gene, and then were cloned into the pVSVXN2-GFP plasmid, respectively. Meanwhile, the GFP gene was replaced with p35/p72. The T7 promoter was inserted at the 5′ end of the infectious cloning site harboring the complete cDNA of the VSV genome, followed by integration of the HDV ribozyme sequence and T7 terminator at the 3′ end. The successfully engineered recombinant plasmids were transcribed using T7 polymerase to generate positive-strand RNA of recombinant VSV. BHK-21 cells were infected with recombinant poxvirus capable of expressing T7 polymerase. Additionally, 293 T cells were co-transfected with plasmids encoding N, P, G, and L proteins of VSV along with an antigenomic copy of the viral genome. Rescue virus supernatants were collected at 96 h post-transfection, and filtered with a 0.22 μm filter after being centrifuged at 1,000 × g for 10 min, which were named VSV-p72 and VSV-p35. Virus clones were plaque-purified, and plaques were amplified on BHK-21 cells as described previously (Ebert et al., 2004). Viral supernatants were harvested upon extensive cytopathic effect by centrifugation at 1,000 × g for 10 min. Aliquots were stored in separate packages at −80°C.

### 3.3 Morphological identification of VSV-p35 and VSV-p72

The volume at 10 μL above purified virus supernatants was dropped onto a copper grid, and the residual liquid was blotted off with filter paper for 10 min. Then 5 μL negative staining with 2% phosphotungstic acid was performed. Finally, the result was observed using an HT7700 transmission electron microscope (TEM, Hitachi High Technologies).

### 3.4 Growth kinetics of VSV-p35 and VSV-p72

The BHK-21 cells were seeded in a 6-well plate with a density of 5 × 10^5^ cells per well and incubated at 37°C for 24 h, and then separately infected with VSV-p72, VSV-p35, and VSV-rwt at a multiplicity of infection (MOI) of 0.01 in triplicate. After being washed with PBS thrice, the cells were covered with DMEM containing 2% FBS. Cell supernatants were collected at 6, 12, 24, 36, 48, 60, 72, and 96 h post-infection (hpi) and stored at −80°C until used. The values of TCID_50_ were determined in BHK-21 cells.

### 3.5 Western blotting (WB)

Total proteins from BHK-21 cells infected with 0.01 MOI of VSV-p72 or VSV-p35 were extracted using ice-cold RIPA lysis buffer at the indicated time (Solarbio, Beijing, China). Briefly, the BHK-21 cells were seeded in a 6-well plate with a density of 1 × 10^6^ cells per well and incubated at 37°C for 24 h, and then infected with VSV-p35 and VSV-p72 with MOI of 0.01, respectively. Cell lysates were centrifuged at 13,400 × g for 8 min to remove cell debris, and the supernatants were boiled for 15 min after being mixed with 4× loading buffer. The equal amounts of protein lysates were subjected to 10% SDS-PAGE polyacrylamide gel and transferred into polyvinylidene difluoride (PVDF) membranes subsequently. The membranes were blocked in PBST with 5% skim milk at room temperature (RT) for 2 h and then incubated overnight at 4°C with HA/His-labeled rabbit polyclonal antibody (diluted at 1:1000). Then the membranes were incubated with horse-radish peroxidase (HRP)-labeled goat anti-rabbit secondary antibody (diluted at 1:5000) for 1 h at RT, respectively. Subsequently, the membranes were visualized using an enhanced chemiluminescence system and a FluorChem E system (ECL, Thermo Scientific, USA) after being washed with PBST thrice.

### 3.6 Indirect immunofluorescence assay (IFA)

The immunofluorescence assay was conducted as follows: BHK-21 monolayer cells that had been infected with 0.01 MOI of VSV-p72, VSV-p35, and VSV-rwt were incubated at 37°C for 24 h. Firstly, the cells were washed five times with PBS after being fixed with 4% paraformaldehyde solution for 20 min at RT. Then, cells were blocked with 5% BSA at RT for 1 h after being permeabilized with 0.25% Triton X-100 for 10 min and then incubated with anti-ASFV swine-positive sera (diluted at 1:50) at 4°C overnight. Next, cells were incubated with the goat anti-pig antibody labeled with fluorescence (diluted at 1:8000) for 1 h at 37°C in darkness, then cells were washed with PBS again five times. Finally, DAPI was added to the cells for 10 min and captured under a fluorescence microscope (Leica, Germany).

### 3.7 Safety assessment of the recombinant virus in BALB/c mice

A total of 200 BALB/c mice were randomly assigned to five different groups (n = 40) and intramuscularly (i.m) inoculated with various doses of VSV-p35, VSV-p72, or a combination of VSV-p35 and VSV-p72, named VSV-p35 + p72. VSV-rwt and PBS were used as control groups. The experimental grouping and immunization doses are shown in Table 1. The survival rate and body weight in mice were monitored and recorded for 28 days post-immunization (dpi). The primary endpoint for safety was the occurrence of adverse blister-related symptoms within 7 days after the vaccination. Collected blood samples from each group of mice on 1, 3, 5, 7, and 9 dpi were analyzed by routine blood examination and viral mRNA quantification. When the viral mRNA levels in the blood reached a peak, mice in each group were euthanized, and fresh hearts, livers, spleens, lungs, and kidneys were harvested. The mRNA levels of VSV in these tissues were measured by RT-qPCR.

**Table 1:** The immunization protocol of the recombinant virus live vector vaccines in BALB/c mice.

Group	Doses (TCID_50_/mouse)	Number of animals	Inoculated route
VSV-p72	5 × 10^6^	8	i.m
	2.5 × 10^6^	8	i.m
	1 × 10^6^	8	i.m
	2.5 × 10^5^	8	i.m
	1 × 10^5^	8	i.m
VSV-p35	5 × 10^6^	8	i.m
	2.5 × 10^6^	8	i.m
	1 × 10^6^	8	i.m
	2.5 × 10^5^	8	i.m
	1 × 10^5^	8	i.m
VSV-p35 + p72	5 × 10^6^	8	i.m
	2.5 × 10^6^	8	i.m
	1 × 10^6^	8	i.m
	2.5 × 10^5^	8	i.m
	1 × 10^5^	8	i.m
VSV-rwt	5 × 10^6^	8	i.m
	2.5 × 10^6^	8	i.m
	1 × 10^6^	8	i.m
	2.5 × 10^5^	8	i.m
	1 × 10^5^	8	i.m
PBS	—	40 (Total)	i.m

### 3.8 Vaccination program in BALB/c mice

A total of 100 six-week-old BALB/c mice were randomly divided into 10 different groups (n = 10) to evaluate the immune potency of the recombinant viruses. The mice were intramuscularly (i.m) or intranasally (i.n) inoculated with 5 × 10^6^ TCID_50_/mouse of VSV-p35, VSV-p72, or VSV-p35 + p72, respectively. VSV-rwt and PBS served as the control groups to compare the immune potency of different recombinant viruses alone or in combination. Sera from the mice in each group was collected weekly after each immunization, and spleen lymphocytes were harvested at 28 dpi.

### 3.9 Humoral immune responses

The specific antibodies against ASFV p72, p30, and p54 in sera were evaluated using an indirect ELISA assay. Briefly, the plate (Costar, Cambridge, MA, USA) was coated with p72 (1 μg/mL), p30 (0.25 μg/mL), or p54 (0.5 μg/mL) in coating buffer (0.05 M carbonate buffer, pH 9.6) (100 μL/well) overnight at 4°C. The plate was blocked with 5% skim milk for 2 h at 37°C and was then incubated with immunized sera samples, anti-ASFV swine-positive sera samples, and negative sera samples (diluted at 1:100) for 45 min at 37°C. Subsequently, the samples were removed and washed four times with PBST, and then HRP-labeled goat anti-mouse IgG, IgG1, or IgG2a (100 μL/well) were incubated in the plate for 45 min at 37°C. Following this, peroxidase substrate TMB was added to each well and incubated for 10–15 min at 37°C. Next, 2 M H_2_SO_4_ was added to stop the reaction, and the OD values at 450 nm were read using a microplate reader (Thermo Fisher Scientific, USA).

### 3.10 Real-time quantitative PCR (RT-qPCR)

Total RNA was extracted from heart, liver, spleen, lung, and kidney tissues and whole blood samples using the TRIzol reagent (Thermo Fisher, United States). RT-qPCR was used to quantify viral RNA by using One Step TB Green PrimeScript™ RT-qPCR Kit II (Takara, Dalian, China), according to the manufacturer’s instructions, using the following cycling program: 42°C for 5 min, 95°C for 10 s, and 40 cycles of 95°C for 5 s and 60°C for 30 s. The primers used in this work were as follows: VSV N gene (F: TAAACATCGGGAAAGCAG, R: GGTTGCCTTTGTATCTACTTG) and β-actin (F: CTGGCACCACACCTTCTACAATGAG, R: TGGCGTGAGGGAGAGCATAGC).

### 3.11 The cytokine detection and lymphocyte proliferation level

The spleen lymphocytes were isolated from immunized mice at 28 dpi and re-suspended with RPMI-1640 medium containing 10% FBS. The spleen lymphocytes were seeded into a 96-well plate at a density of 2 × 10^6^ cells per well and were then stimulated by adding various concentrations (2.5 × 10^4^, 1 × 10^5^, 4 × 10^5^ HAD_50_/well) of heat-inactivated ASFV. Concanavalin A, unstimulated cells, and RPMI-1640 medium were set as positive, negative, and blank controls, respectively. The level of cytokines in supernatants in each well was detected after the plate was incubated for 72 h at 37°C. The Cell Counting Kit-8 assay solution (CCK8, Dojindo, Japan) was added and incubated for 4 h at 37°C. Finally, the absorbance of OD_450_ was measured. The calculated formula is as follows: the stimulation index SI = (experimental group OD_450_ - blank control OD_450_) / (negative control OD_450_ - blank control OD_450_).

### 3.12 Detection of T lymphocyte phenotype by flow cytometry assay

The spleen lymphocytes were isolated from the vaccinated BALB/c mice using Ficoll Plus 1.077 (Solarbio, Beijing, China). The density of cells was adjusted into 2 × 10^7^/ml with RPMI-1640 medium containing 10% FBS. The spleen lymphocytes were co-incubated with heat-inactivated ASFV (10^5^ HAD_50_) in 96-well plates (100 μL/well) for 40 h at 37°C in 5% CO_2_, then 1.7 μg/mL of monensin was added to each well to culture for 8 h. The cell supernatants were stained with APC-CD3, FITC-CD4, and Phycoerythrin-CD8 (BD Biosciences, San Diego, USA) for 30 min at 4°C in darkness. The permeabilization solution was added and incubated for 30 min at 4°C in darkness, after which cells were washed with PBS twice. Then, supernatants were aspirated and cells were washed by adding 1 mL of BD perm/wash™ buffer for 5 min in darkness. The cells were re-suspended in the 100 μL PBS, with 2 μL PerCP-Cyanine5.5 IL-2 and IFN-γ 450 mAb, and cells were incubated for 30 min at 4°C in darkness. The cells were washed and re-suspended with PBS, then cell populations were analyzed by flow cytometry (Beckman Coulter, Brea, CA, United States).

### 3.13 Histopathological and immunohistochemistry analysis

The mice were euthanized at 28 dpi, and fresh hearts, livers, spleens, lungs, and kidneys were collected and fixed with 10% neutral buffered formalin. Tissues were paraffin-embedded, and 5 μm sections were subsequently stained with hematoxylin and eosin. Unimmunized mice were stained in parallel and used as negative controls. The unstained sections were firstly dewaxed with xylene three times and then rehydrated by ethanol gradient (100% EtOH, 70% EtOH, 50% EtOH, 30% EtOH) for 5 min, respectively, followed by washing three times with distilled water for 3 min. Then, Tris/EDTA buffer pH 9.0 was added to repair the antigen in a microwave oven for 5 min. Next, 3% hydrogen peroxide was added to inactivate endogenous peroxidase for 10 min and washed three times with tris-buffered saline (TBS) for 5 min. After this, the sections were blocked with 10% normal goat serum for 1 h at RT and incubated with the anti-VSV-G tag antibody (Abcam, Cambridge, UK) at 1:2000 dilution overnight at 4°C, and washed three times with TBS for 3 min. The sections were incubated with HRP-labeled secondary antibodies for 30 min at RT and then washed three times with TBS for 3 min. Finally, tissue sections were incubated with diaminobenzidine (DAB) for 5 min, washed with distilled water, counterstained with hematoxylin for 3 min, dehydrated, and visualized using a Nikon microscope (Eclipse Ci-L, Japan) equipped with an Olympus DP71 color camera using 200× magnification.

### 3.14 Neutralization assay

The sera samples from immunized BALB/c mice were first heat-inactivated for 30 min at 56°C. The covered PAM cells in 24-well plates were incubated with ASFV CN/SC/19 (200 HAD_50_) and heat-inactivated sera (dilution 1:4, 1:8, 1:16, and 1:32) for 1 h at 37°C. Subsequently, the cells were cultured with RPMI-1640 supplemented with 5% FBS for 72 h after being washed with PBS. The ASFV genomes in each well were extracted using a SteadyPure virus DNA/RNA extraction kit (Accurate Biology, China), and the recombinant plasmid of the B646L gene was amplified by RT-qPCR as described previously (Miao et al., 2023). The virus copies in each sample were calculated according to the CT value, and the established standard curve to determine the virus neutralization rate used the following formula:

$\begin{array}{l} \mathrm{Neutralization}\;\left(\%\right)=100-100\times B646L\;\mathrm{gene copy number of}\\ \mathrm{immune sera}/B646L\;\mathrm{gene copy number of}\;pre-\mathrm{immune sera}\text{.} \end{array}$

### 3.15 Statistical analysis

All experiments were repeated thrice, and the data in the figures were presented as mean ± standard error of the mean (SEM). Statistically significant differences in the anti-p72, anti-p30, anti-p54 humoral, and cellular immune responses between groups of immunized mice were evaluated by two-way ANOVA with Bonferroni’s multiple comparison test. *p* < 0.05 was considered statistically significant, and *p* < 0.001 was considered to be extremely significant.

## 4 Results

### 4.1 Construction and characterization of VSV-p72 and VSV-p35

**Figure 1:**
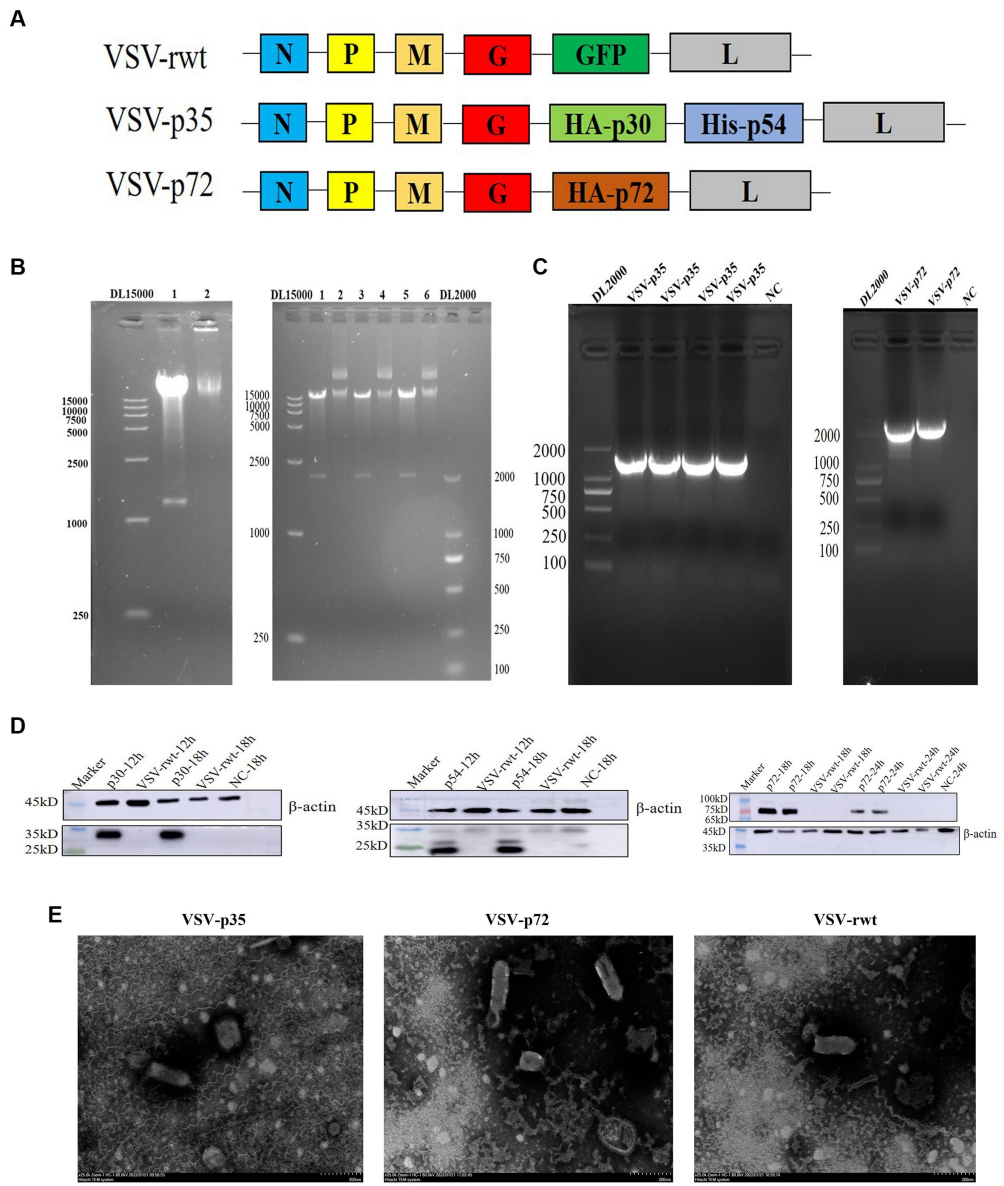
Construction of the recombinant viruses (VSV-p72 and VSV-p35). **(A)** The schematic representation of the recombinant viruses. **(B)** The identification of recombinant plasmids with Xho I and Nhe I restriction enzymes, 15 kb in size was the pVSVXN2-GFP vector fragment, and the other was the target gene fragment, 1980 bp and 1,233 bp in size, respectively. 1, 3, 5: the enzyme digestion plasmid; 2, 4, 6: the uncleaved plasmid. **(C)** PCR amplification of the target genes. **(D)** The target proteins expression of VSV-p35 and VSV-p72 in infected BHK-21 cells. **(E)** The morphology of the recombinant viruses under transmission electron microscopy; the scale bar was equivalent to 200 nm.

The codon-optimized B646L, CP204L, and E183L gene sequences of ASFV were cloned into the pVSVXN2-GFP vector at the G–L junction in the VSV genome to construct recombinant plasmids, respectively (Figure 1A). To identify the recombinant plasmids, the recombinant plasmids were digested with *Xho I* and *Nhe I* restriction enzymes, and two bands were generated: one was the pVSVXN2-GFP vector fragment, about 15 kb in size, and the other was the target gene fragment, about 1980 bp of B646L and 1,233 bp of CP204L-E183L in size (Figure 1B). VSV-p72 and VSV-p35 were packaged using the reverse genetic system in HEK293T cells and produced a significant cytopathic effect in BHK-21 cells. RT-PCR results showed that the bands of VSV-p72 and VSV-p35 in the cell supernatants were consistent with the size of the target genes (Figure 1C). To verify whether recombinant viruses VSV-p35 and VSV-p72 can express the p30, p54, and p72 proteins of the ASFV in the BHK-21 cells, respectively, the WB assay was executed. The results of WB showed that the p30, p54, and p72 proteins were detected at 18 hpi, while the expression of the target protein was not detected in VSV-rwt and the non-infected cell control group, as shown in Figure 1D. The ultrastructural morphology of recombinant viruses was observed with transmission electron microscopy. The results showed that reassembled virus particles were a typical bullet shape and approximately 190 nm in length (Figure 1E). These results indicated that VSV-p72 and VSV-p35 were successfully packaged in BHK-21 cells.

### 4.2 Genetic stability of VSV-p72 and VSV-p35

**Figure 2:**
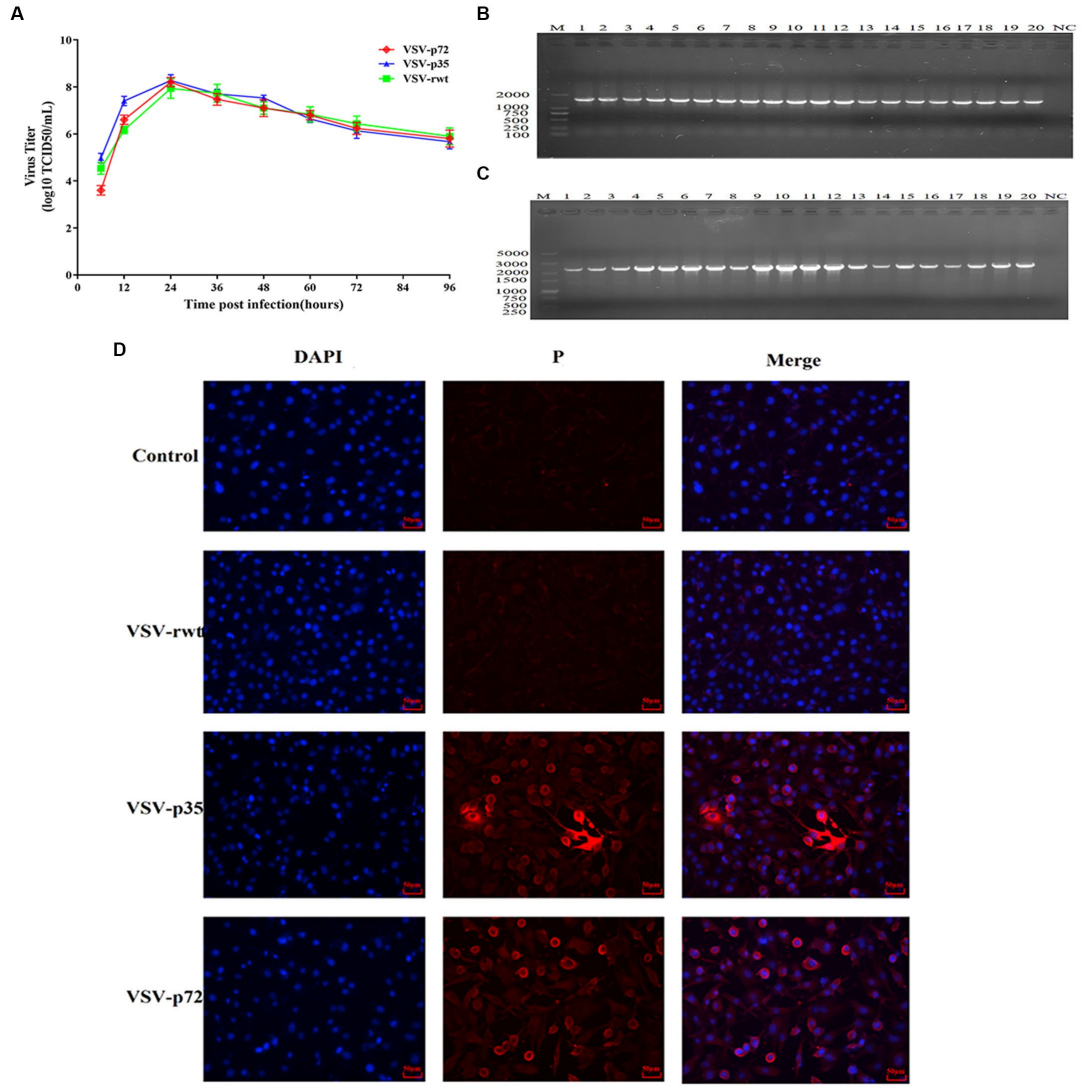
Genetic stability of recombinant viruses. **(A)** The growth properties of VSV-p35 and VSV-p72 were inoculated into BHK-21 cells at an MOI of 0.001. **(B,C)** RT-PCR amplification results in cell supernatants by serially passaging the recombinant viruses for 20 generations. **(D)** The results of IFA analysis on target proteins extracted from BHK-21 cells infected with VSV-p35 and VSV-p72 at the 20th passage.

Although the insertion of the exogenous protein renders the package of the recombinant virus less efficient, our results showed that the highest viral titer of the recombinant virus VSV-p35 could reach 10^8.5^ TCID_50_/mL in BHK-21 cells at 24 hpi (Figure 2A). In the present study, the BHK-21 cells were infected with VSV-p72/VSV-p35 (MOI = 0.001), and the genetic stability of recombinant viruses was evaluated by serially passaging the viruses for 20 generations. RT-PCR results showed that the supernatants in generations 1–20 of viruses could amplify a band similar to the size of the target gene, while there was no objective band in the control group (Figures 2B,C). Immunofluorescence results demonstrated that specific fluorescence signals were detected in recombinant viruses infected cells but not in control cells and VSV-rwt infected cells (Figure 2D). Taken together, these results confirmed that ASFV p30, p54, and p72 protein can be expressed efficiently and stably in the recombinant viruses.

### 4.3 Pathogenicity of VSV-p72 and VSV-p35 In BALB/c mice

**Figure 3:**
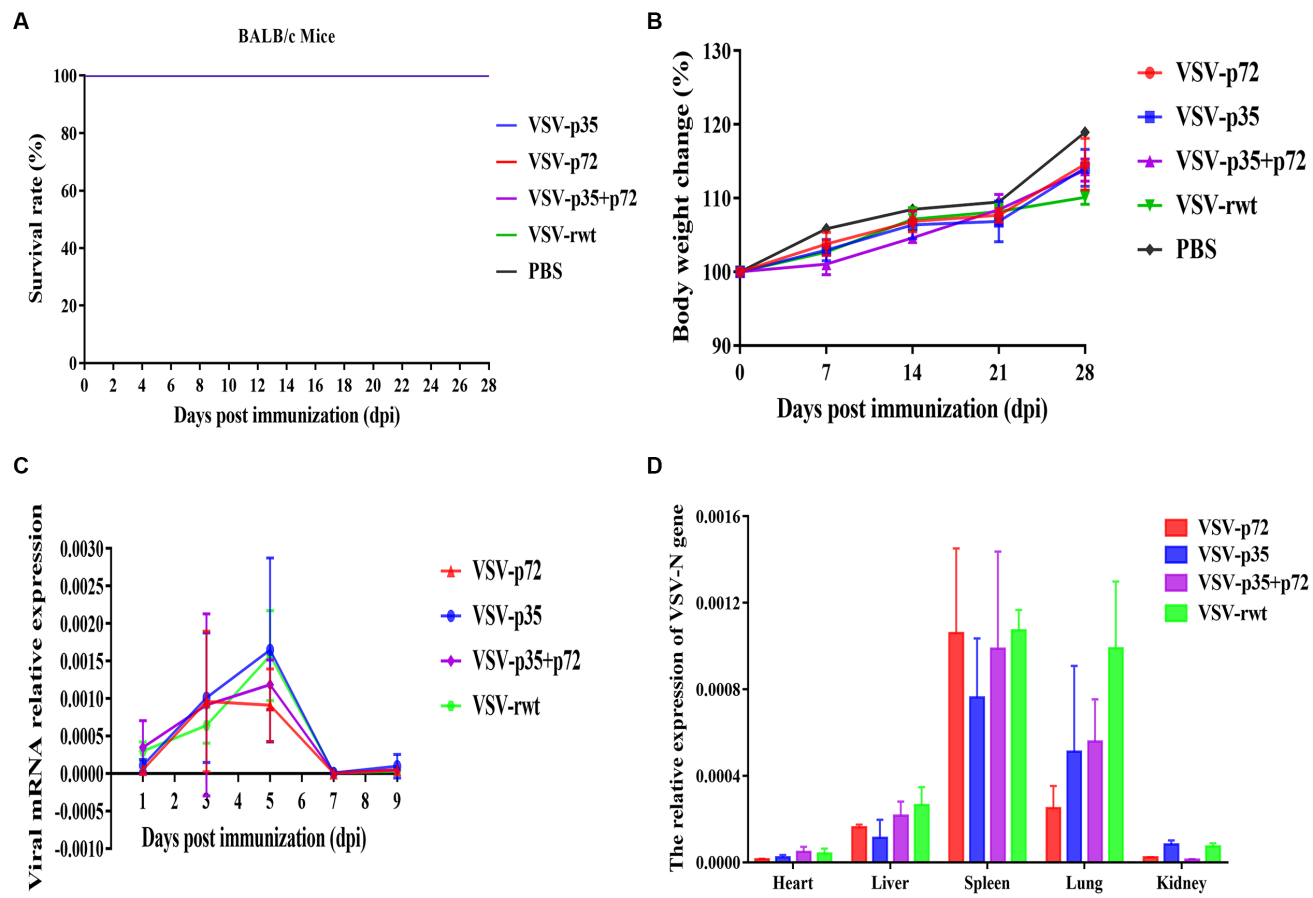
Safety evaluation of recombinant viruses in BALB/c mice. **(A)** The survival rate of mice that had been inoculated with recombinant viruses. **(B)** The body weight change of mice that had been inoculated with recombinant viruses. **(C,D)** The relative expression of the VSV N gene in blood, heart, liver, spleen, lung, and kidney samples of BALB/c mice inoculated with recombinant viruses, respectively.

**Figure 4:**
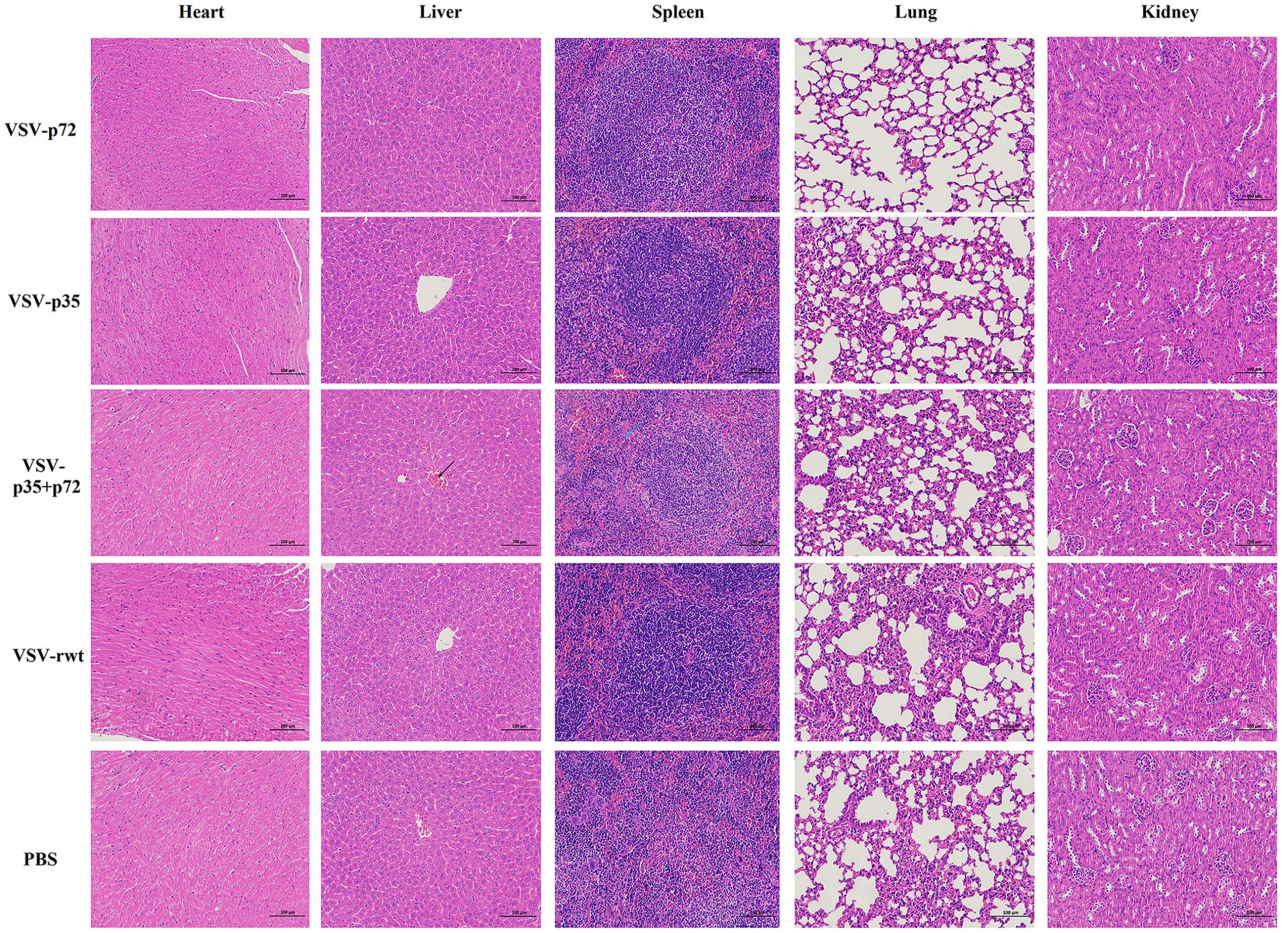
HE staining to analyze the histopathologic changes in the hearts, livers, spleens, lungs and kidneys of BALB/c mice inoculated with recombinant viruses at 28 dpi. VSV-rwt and PBS served as control groups (bar = 100 μm, 200×). Black arrow: central venous congestion; blue arrow: a small amount of granulocyte infiltration; HE, hematoxylin–eosin.

**Figure 5:**
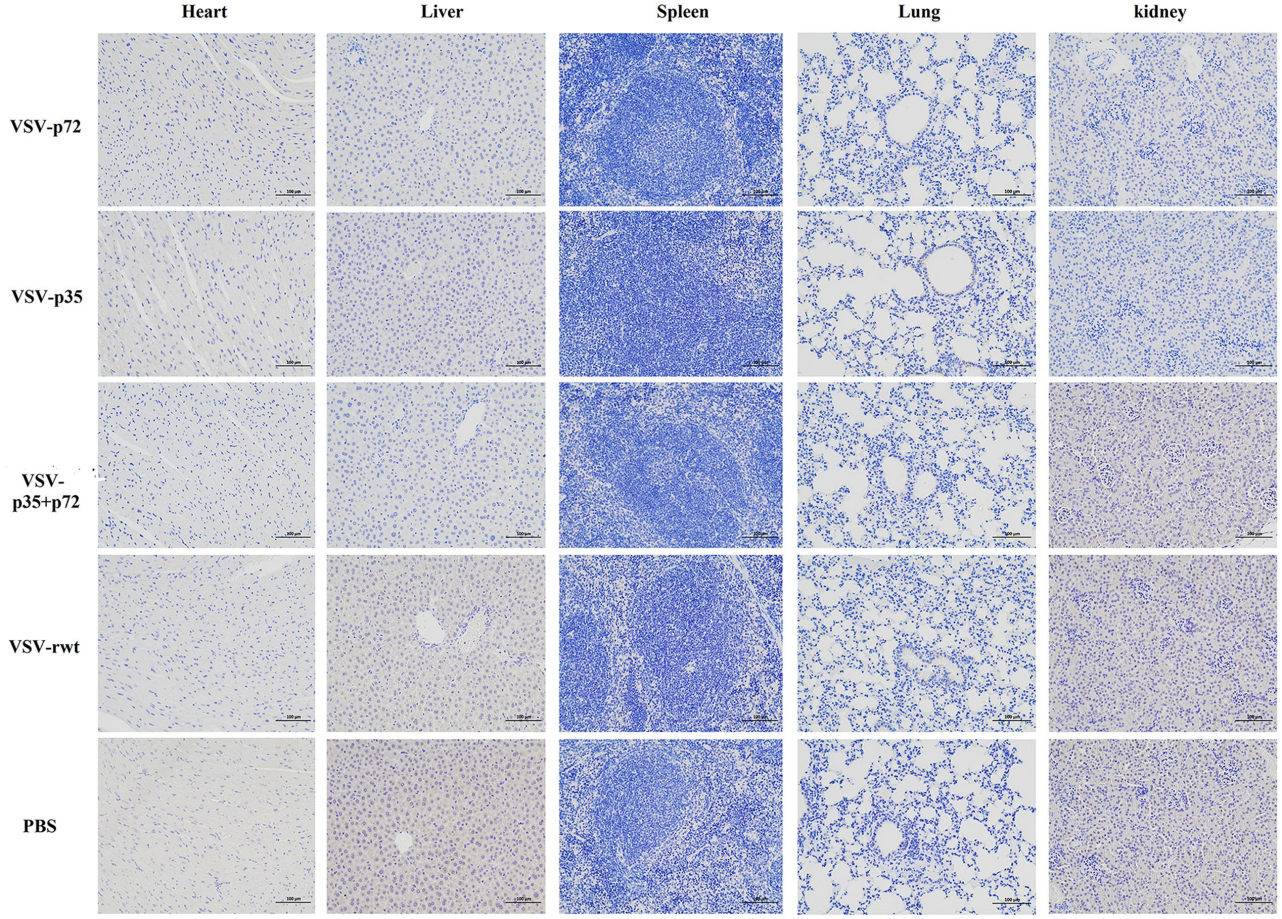
Immunohistochemical detection of VSV G protein in the hearts, livers, spleens, lungs, and kidneys of BALB/c mice inoculated with recombinant viruses at 28 dpi. VSV-rwt and PBS served as control groups (bar = 100 μm, 200×).

BALB/c mice were intramuscularly inoculated with VSV-p35, VSV-p72, and VSV-p35 + p72 at a dose from 1 × 10^5^ to 5 × 10^6^ TCID_50_ per mouse to evaluate the safety of the recombinant viruses. The VSV-rwt and PBS served as control groups. The results showed that BALB/c mice infected with VSV-p35, VSV-p72, and VSV-p35 + p72 all survived without adverse clinical symptoms until the end of the experiment, and body weight loss of mice did not occur (Figures 3A,B). In addition, viral mRNA levels in blood, heart, liver, spleen, lung, and kidney samples were detected at 1, 3, 5, 7, and 9 dpi, respectively. The results showed that the viral mRNA levels in the immunized groups and VSV-rwt control group could be detected in a small amount at 3 dpi and 5 dpi but could not be detected at any other time, with no significant difference between them at the same time point (*p* > 0.05, Figure 3C). There was no virus detected in other tissues except for a small amount of VSV mRNA in the spleens and lungs (Figure 3D). These results indicated that the residual amount of the recombinant viruses decreased in the blood and tissues of mice with the passage of immune time. Histopathological results showed that all immunized groups, as well as the VSV-rwt and PBS control groups, were normal, with no pathological changes in the hearts, lungs, or kidneys. However, minor lesions were observed in the spleen and liver of the VSV-p35 + p72 group, including central venous congestion in the liver, with a small amount of granulocyte infiltration in the spleen (Figure 4). The immunohistochemical results showed that compared with the VSV-rwt control group, VSV G protein expression was not detected in the hearts, livers, spleens, and kidneys of all immunized mice, except for a small amount of VSV G protein detected in the lung of the VSV-p35 group (Figure 5).

**Figure 6:**
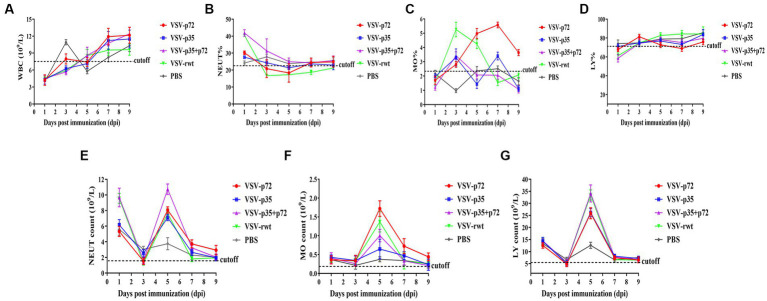
Blood routine analysis in mice that had been inoculated with recombinant viruses. **(A)** The total white blood cell count (WBC). **(B–D)** The percentage of neutrophils (NEUT), monocytes (MO), and lymphocytes (LY), respectively. **(E–G)** The total number of neutrophils, monocytes, and lymphocytes in turn.

A routine blood examination was conducted to analyze the effect of recombinant viruses on mice. The results showed that the total number of white blood cells, monocytes, and neutrophils in mice that had been inoculated with the recombinant viruses showed a decreasing trend, and an increase in lymphocyte count at the early stages of infection appeared (as shown in Figures 6A–G). These changes were consistent with the regular pattern of virus infection, indicating that the recombinant viruses had no obvious impact on the mice. Based on these results, the recombinant viruses VSV-p72 and VSV-p35 preliminarily showed excellent safety in BALB/c mice.

### 4.4 Determination of the optimal inoculation dose

**Figure 7:**
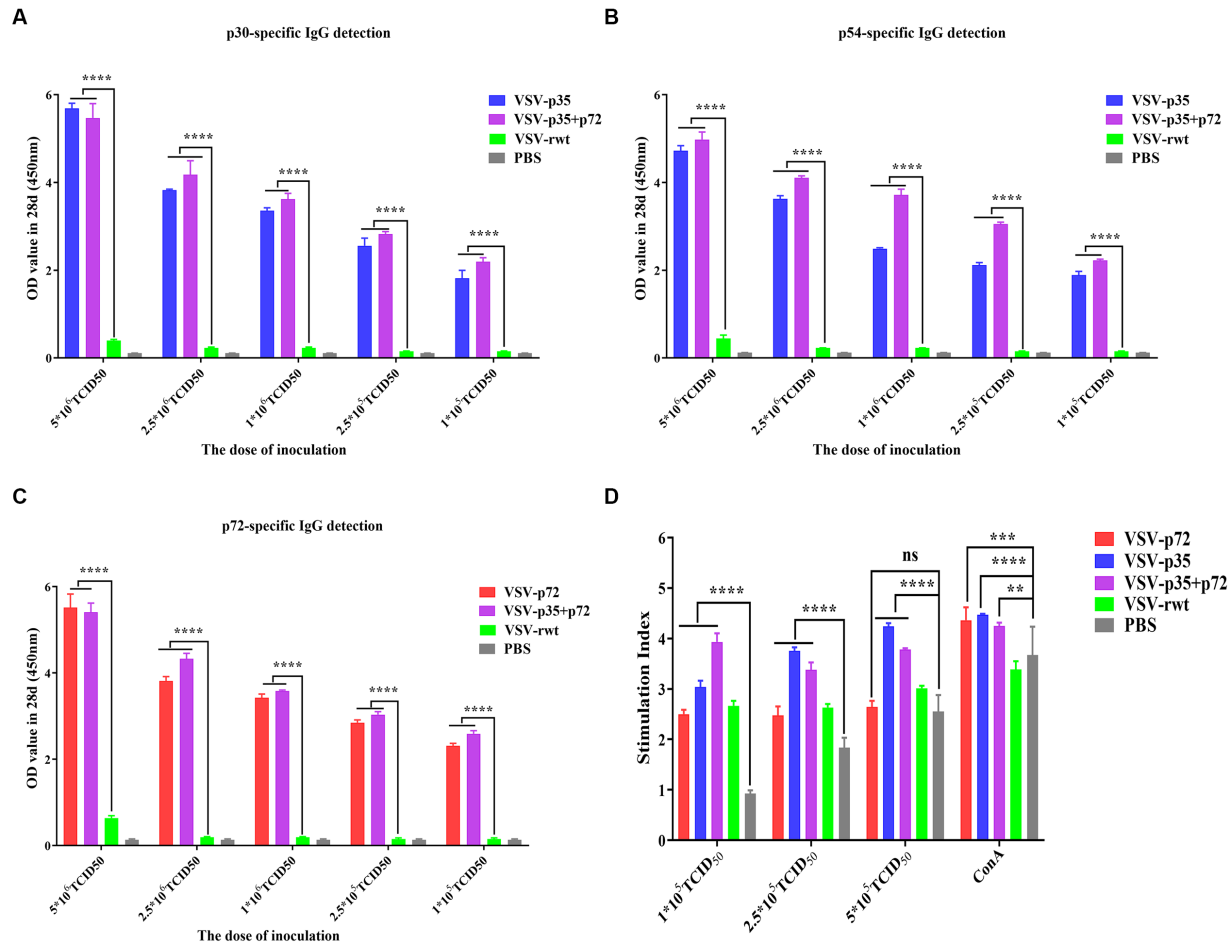
Evaluation of immunogenicity in mice immunized with different doses of recombinant viruses. **(A–C)** The levels of IgG to p30, p54, and p72. **(D)** The proliferation of T lymphocytes from immunized mice stimulated with different doses (1 × 10^5^, 2.5 × 10^5^, and 5 × 10^5^ TCID_50_/mL) of the recombinant viruses. The data are presented as the means ± SD. Statistical significance is denoted by ns = *p* > 0.05, *p < 0.05, **p < 0.01, and ***p < 0.001.

In order to further determine the optimal inoculation dose, the specific IgG antibody levels at different immune times were detected with an indirect ELISA assay. As shown in Figures 7A–C, p30, p54, and p72 specific IgG levels showed no significant difference in VSV-p35, VSV-p72, and VSV-p35+ p72 immunized groups at 7 dpi (*p* > 0.05). However, mice that had been immunized with VSV-p35, VSV-p72, or VSV-p35 + p72 produced higher levels of IgG than mice that had been immunized with VSV-rwt and PBS groups at 21 dpi and 28 dpi (*p* < 0.01). The specific IgG levels in VSV-p35, VSV-p72, and VSV-p35 + p72 groups increased in a dose-dependent manner. Among them, the VSV-p35 + p72 group produced the highest specific IgG levels that targeted p30, p54, and p72 at different immune times, and there were no specific IgG levels that targeted p30, p54, and p72 in all VSV-rwt and PBS control groups.

The lymphocyte proliferation levels of immunized groups were detected by the CCK8 reagent. The results showed that lymphocyte proliferation levels were increased significantly both the immunized groups and the VSV-rwt group after being stimulated with different doses (1 × 10^5^, 2.5 × 10^5^ and 5 × 10^5^ TCID_50_/mL) of the recombinant viruses (*p* < 0.001). However, the proliferation levels in the immunized groups were significantly higher than the levels in the PBS control group (*p* < 0.01), and the proliferation levels in higher dose stimulation groups reached the highest level (Figure 7D). The above results indicate that the spleen lymphocytes in mice that had been immunized with recombinant viruses exhibited good proliferation and activation capacity by being stimulated with the recombinant viruses.

### 4.5 Effect of single-dose intramuscular or intranasal immunization with recombinant virus on IgG antibody response

**Figure 8:**
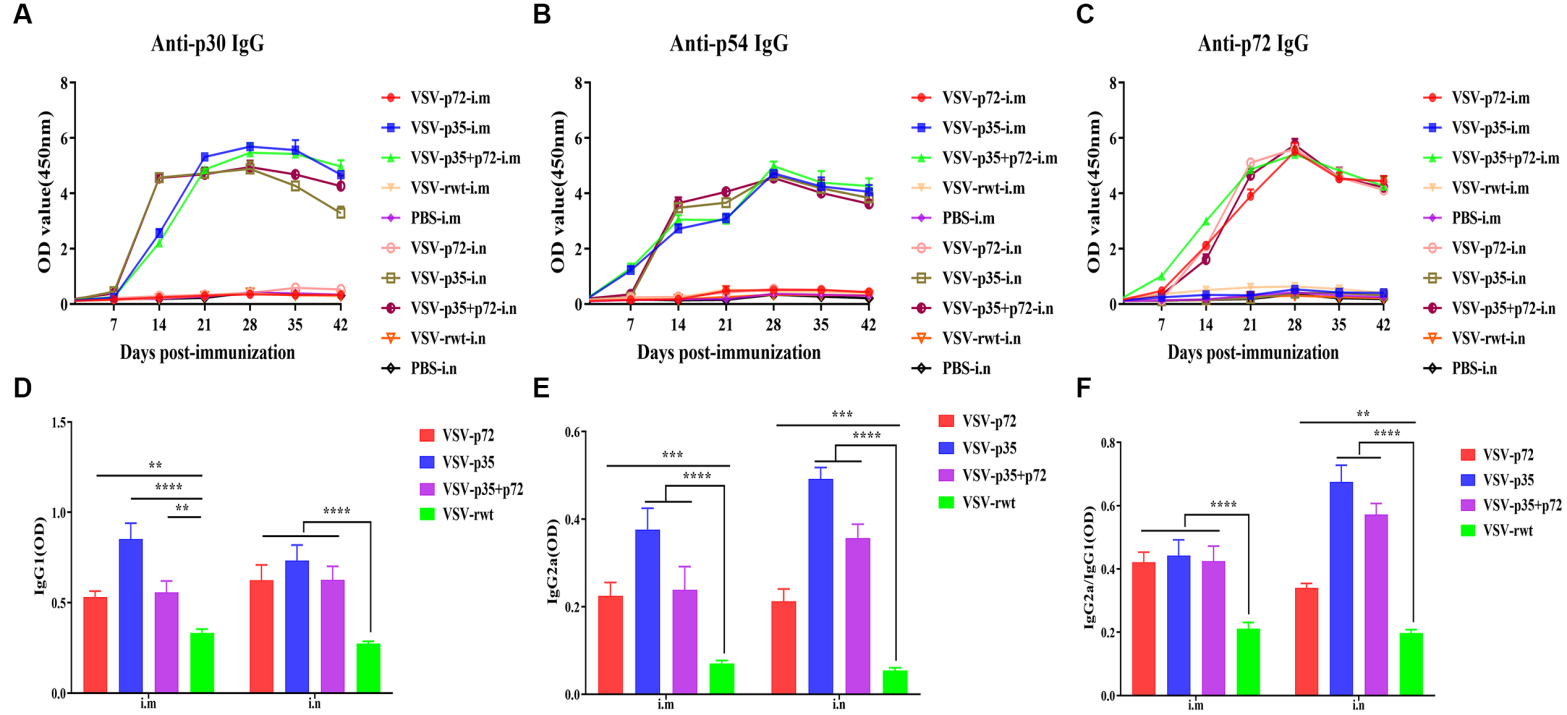
The antibody levels in sera. **(A–C)** The specific IgG levels against ASFV to p30, p54, and p72 in each group. **(D,E)** The levels of IgG1 and IgG2a in sera, respectively. **(F)** The calculated ratio of IgG2a/IgG1. The data are presented as the means ± SD. Statistical significance is denoted by ns = *p* > 0.05, *p < 0.05, **p < 0.01, and ***p < 0.001.

The indirect ELISA method was used to detect the ASFV-specific IgG levels in sera. The results showed that specific IgG antibodies against ASFV were not detected in pre-immunized mice sera, but specific IgG antibodies to p30, p54, and p72 were detected at 7 dpi and subsequently began to rise at 14 dpi and peaked at 28 dpi, where high levels of antibodies were maintained for 42 days continuously (Figures 8A–C). There was no statistical difference between the i.m and i.n immunized groups at any time (*p* > 0.05), but it was significantly higher than the VSV-rwt and PBS groups (*p* < 0.001). In addition, the antibody subtype analysis results showed that IgG1 and IgG2a levels in the VSV-p35, VSV-p72, and VSV-p35 + p72 groups were overall higher than in the VSV-rwt group (*p* < 0.001), and the IgG1 and IgG2a levels in the VSV-p35 group were significantly higher compared to the VSV-p72 and VSV-p35 + p72 groups (*p* < 0.05) (Figures 8D,E). The IgG2a/IgG1 ratio in immunized groups was significantly higher than in the VSV-rwt group (*p* < 0.001) (Figure 8F). The above data indicates that a single dose of recombinant virus vaccine prototypes triggered a robust, specific IgG response, which was maintained for 42 days in mice, including in IgG1 and IgG2a subtypes.

### 4.6 Effect of the recombinant virus on cytokine level and lymphocyte proliferation

**Figure 9:**
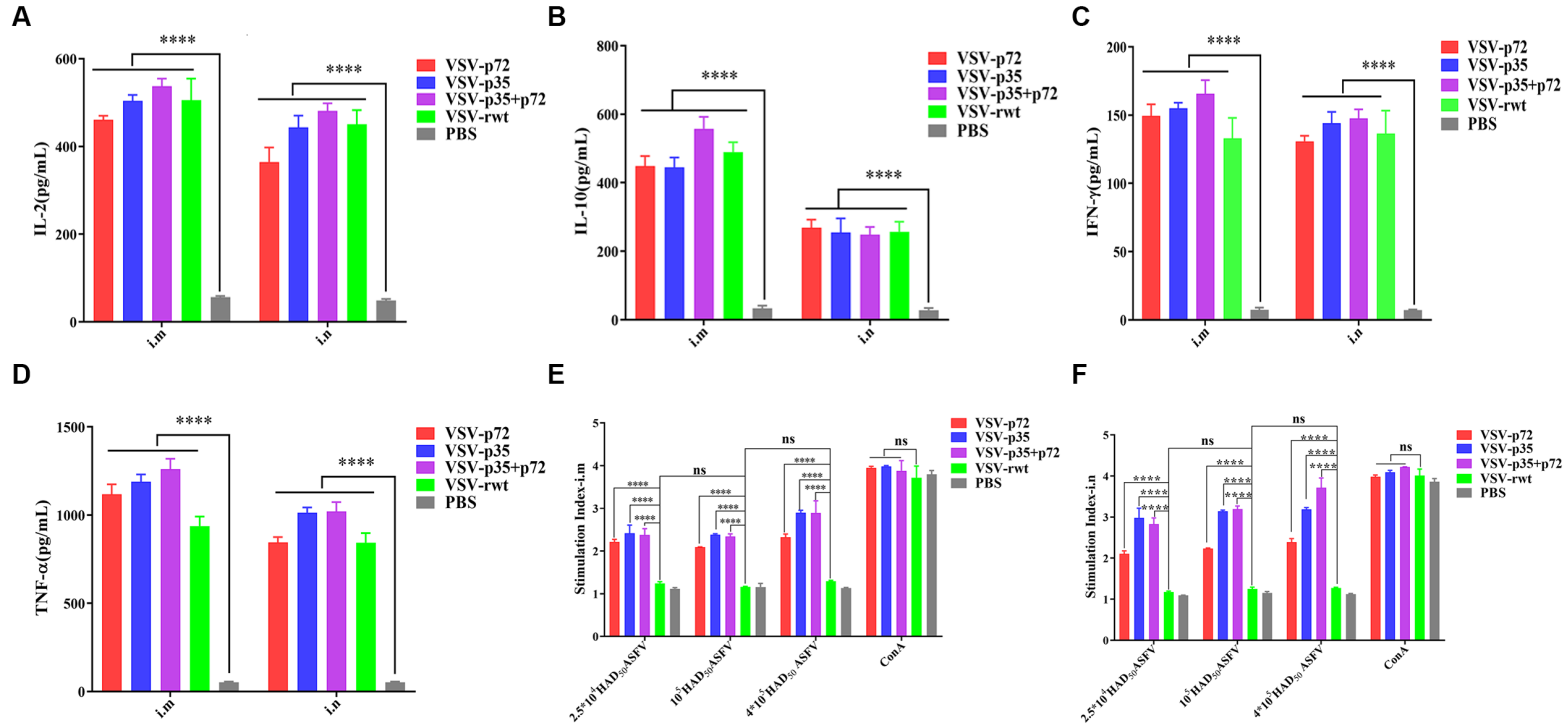
The cytokine secretion and T lymphocyte proliferation in spleen lymphocytes from mice immunized with the recombinant viruses or PBS. **(A–D)** The IL-2, IL-10, IFN-γ, and TNF-α levels secreted in spleen lymphocytes from i.m or i.n immunized mice. **(E,F)** The proliferation of T lymphocytes from i.m or i.n immunized mice stimulated by different doses (2.5 × 10^4^, 1 × 10^5^, 4 × 10^5^ HAD_50_/well) of heat-inactivated ASFV. The data are presented as the means ± SD. Statistical significance is denoted by ns = *p* > 0.05, *p < 0.05, **p < 0.01, ***p < 0.001.

As shown in Figures 9A–D, the IL-2, IL-10, IFN-γ, and TNF-α secretion in spleen lymphocytes of immunized mice was significantly higher than in the PBS group (*p* < 0.001). However, there was no significant difference in the i.m immunized groups compared to the i.n immunized groups (*p* > 0.05). These results suggest that recombinant virus vaccine prototypes may activate cellular immune responses. In order to further determine the cellular immune responses induced by the recombinant virus vaccine prototypes in mice, the proliferation effect of spleen lymphocytes was monitored using the CCK8 assay. The results showed that the lymphocyte proliferation levels in immune groups significantly exceeded those in the VSV-rwt and PBS groups after being treated with heat-inactivated ASFV stimulation (*p* < 0.001). However, there was no significant difference between the routes of i.m vaccination and i.n vaccination under the different doses of heat-inactivated ASFV stimulation (*p* > 0.05, Figures 9E,F). These results indicate that the spleen lymphocytes in immunized mice exhibited good proliferation and activation ability with inactivated ASFV stimulation.

### 4.7 T cell immune response of recombinant virus

**Figure 10:**
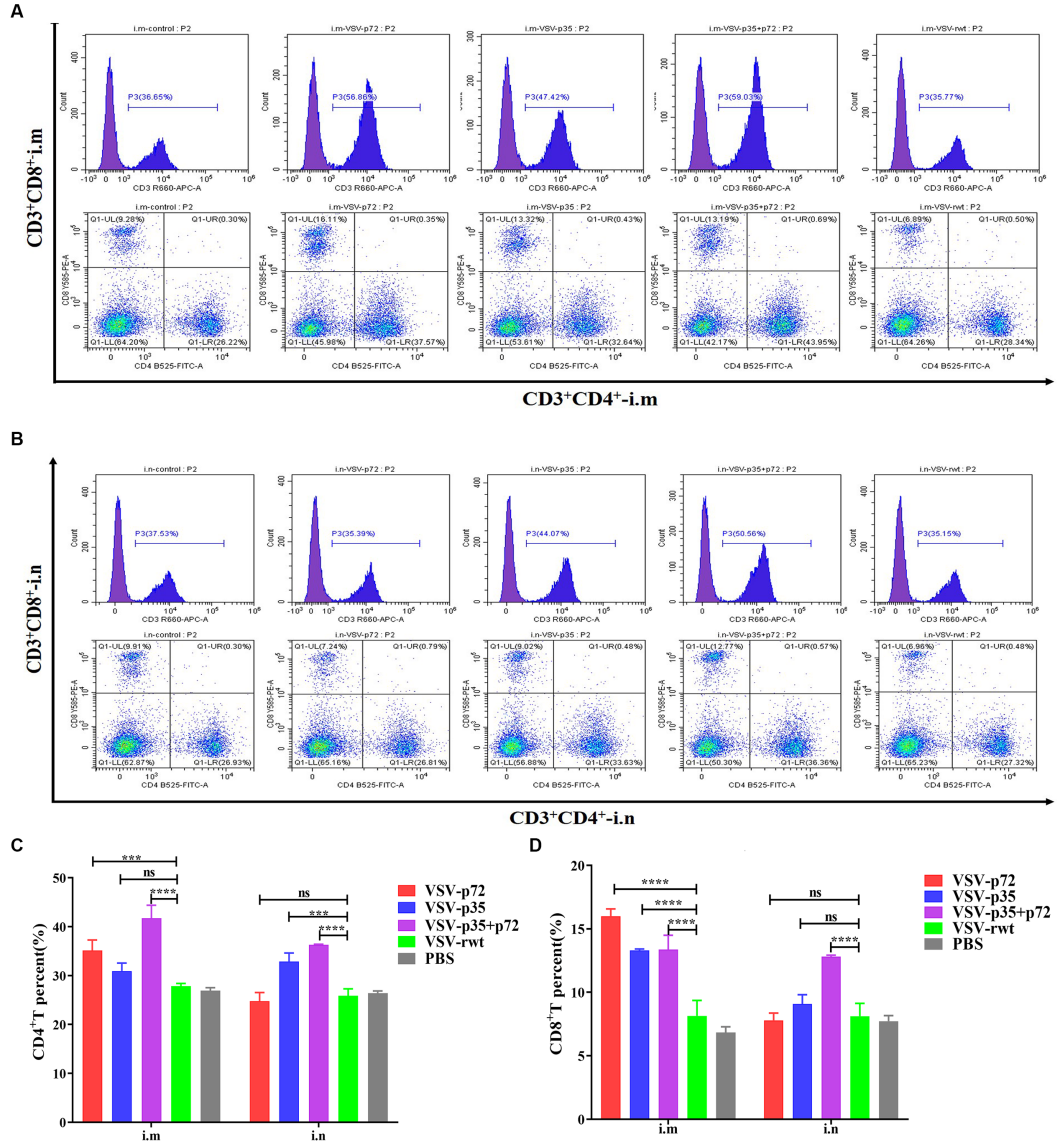
Spleen lymphocyte typing of immunized mice. **(A,B)** The percentage of CD4^+^ and CD8^+^ T in spleen lymphocytes from i.m or i.n immunized mice, respectively. **(C,D)** The statistical results of CD4^+^ and CD8^+^ T in spleen lymphocytes. The data are presented as the means ± SD. Statistical significance is denoted by ns = *p* > 0.05, *p < 0.05, **p < 0.01, ***p < 0.001.

**Figure 11:**
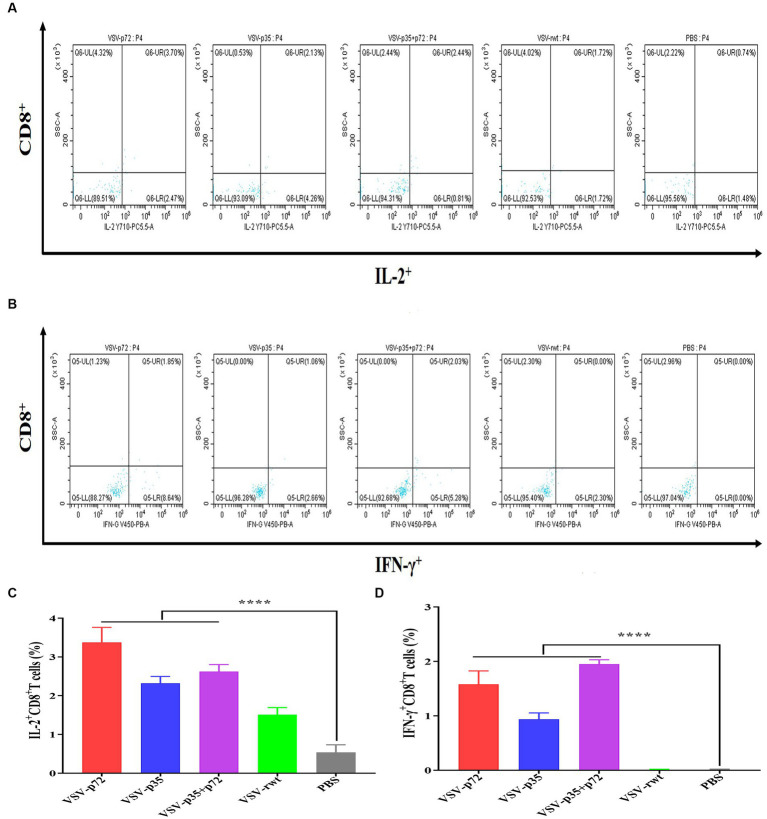
Intracellular cytokine expression in spleen lymphocytes from immunized mice stimulated by inactivated ASFV or PBS. **(A,C)** The percentage and statistical results of IL-2^+^ CD8^+^ T cells in CD8^+^ T cells, respectively. **(B,D)** The percentage and statistical results of IFN-γ^+^ CD8^+^ T cells in CD8^+^ T cells. The data are presented as the means ± SD. Statistical significance is denoted by ns = *p* > 0.05, *p < 0.05, **p < 0.01, ***p < 0.001.

The effect of recombinant virus vaccine prototypes on spleen lymphocytes in mice was evaluated, and a ratio of CD3^+^ CD4^+^ and CD3^+^ CD8^+^ in spleen was detected by flow cytometry. The levels of CD4^+^T lymphocytes in mice showed no statistical difference between the i.m immunized and i.n immunized groups (*p* > 0.05). However, the levels of CD8^+^ T lymphocytes in the i.m-immunized groups were significantly higher than in the i.n-immunized groups (*p* < 0.001, Figures 10A–D). These results indicate that mice vaccinated with recombinant viruses by i.m immunized and i.n immunized routes triggered the activation of T cell responses, and the effect of the i.m immunized route to cellular immune response was better than the i.n immunized route. In addition, IL-2^+^ CD8^+^ T cells and IFN-γ^+^ CD8^+^ T cells in spleen lymphocytes were measured. Compared to control groups, the spleen lymphocytes in VSV-p35, VSV-p72, and VSV-p35 + p72 groups stimulated by inactivated ASFV showed a significant increase in the proportion of IL-2^+^ CD8^+^ T cells and IFN-γ^+^ CD8^+^ T cells, as shown in Figures 11A–D (*p* < 0.001). The above results indicate that spleen lymphocytes from mice that had been immunized with recombinant viruses exhibited an excellent activation ability from ASFV stimulation and a functional T lymphocyte response.

### 4.8 Neutralizing effect of recombinant virus vaccine prototypes

**Figure 12:**
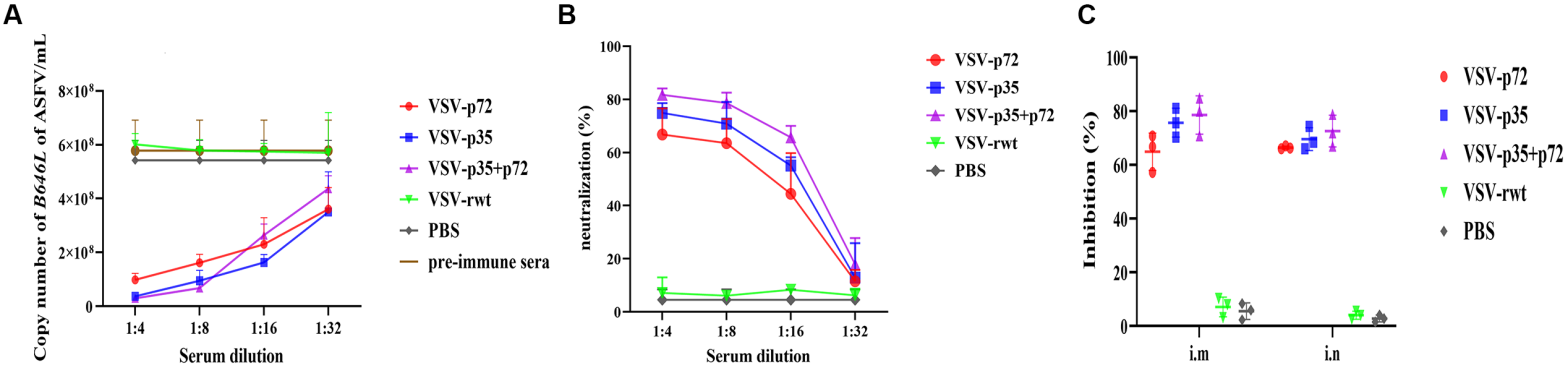
The ability of sera from immunized BALB/c mice to neutralize ASFV infection *in vitro*. ASFV CN/SC/19 virus strain pre-incubated with pre-immune sera as a control or immune sera collected at 28 dpi after vaccination was used to infect PAM cells. The copy number of the B646L gene was determined by qPCR to measure the neutralization efficiency of the recombinant viruses after being infected for 72 h. **(A,B)** The copy number of the B646L gene and neutralization rate of immunized BALB/c mice sera at different dilutions after incubation with ASFV. **(C)** The neutralization rate of immunized BALB/c mice sera at 1:8 diluting after incubation with ASFV.

To analyze the ASFV neutralizing activity of immune sera, ASFV CN/SC/19 (200 HAD_50_) was co-incubated with heat-inactivated immune sera for 72 h to detect the neutralizing ability. As shown in Figures 12A–C, compared with the VSV-rwt and PBS control groups, the immune sera of immunized groups significantly reduced the ASFV genome copy number (*p* < 0.001), indicating the inhibition of ASFV proliferation in PAMs. In contrast, the sera from the PBS control group and pre-immunization did not show the ability to reduce the copy number of the ASFV genome. Based on these results, the percentage of neutralizing ASFV in the immune sera of each group was calculated. The average neutralization rates of VSV-p72, VSV-p35, and VSV-p35 + p72 were 64.89, 75.86, and 78.58%, respectively, and there was no significant difference between the i.m and i.n immunized groups (*p* > 0.05).

## 5 Discussion

ASF is a viral disease that results in high incidence and mortality rates among domestic pigs and wild boars, causing significant economic losses in the pig industry. Given the lack of effective therapeutic drugs or commercial vaccines, it only is controlled through strict biosafety protocols, quarantine measures, and culling infected animals in epidemic areas (Ge et al., 2018; Zhao et al., 2019; Gladue et al., 2023; Zhao et al., 2023). Currently, vaccination is considered the most effective measure for preventing and controlling ASF. However, despite multiple attempts to develop vaccines against ASFV, significant success has yet to be achieved (Roberts et al., 1998; O'Donnell et al., 2017; Franzoni et al., 2018; Gallardo et al., 2018; Pérez-Núñez et al., 2019). Viral live vector vaccines have been a research focus due to their ability to elicit strong humoral and cellular immune responses in the host. VSV is capable of accommodating longer exogenous fragments, expressing exogenous proteins stably and efficiently, growing at high titer in most mammalian cells, and can be easily prepared in large quantities (Rose et al., 2000). The VSV is sensitive to IFN and can be rapidly eliminated from the host through the induction of a robust immune response. Studies have demonstrated that live attenuated recombinant VSV expressing the HIV nuclear proteins and the Lassa viral glycoprotein protected against pathogenic viruses, which supports the suggestion that VSV as a viral vector that expresses exogenous antigens is favorable for a safe and immunogenic response against highly pathogenic strains (Rose et al., 2001; Geisbert et al., 2005). The VSV as a vaccine vector was safe in pigs, a result that has been supported by previous reports where the VSV vector-based vaccine has produced an effective and specific immune response against porcine epidemic diarrhea virus and swine influenza virus in pigs (Ricklin et al., 2016; Ke et al., 2019). Furthermore, De Wit et al. observed that the inoculation of pigs with rVSVwt and rVSV∆G/EBOVGP did not result in apparent clinical manifestations, and viral shedding was minimal. However, it was noted that rVSV∆G/EBOVGP exhibited replicative ability in pigs (De Wit et al., 2015). Interestingly, the pigs inoculated with VSV-EBOV did not exhibit any apparent vesicular clinical symptoms, and horizontal transmission was not induced in swine (Geisbert et al., 2008; Suder et al., 2018; Matz et al., 2019; Morozov et al., 2021). These findings provided safety evidence for promoting the development and characterization of numerous other candidate vaccines against emerging and pathogenic viruses. Taking the above results into consideration, VSV as a vaccine vector is safe and could be applied to develop a novel ASF vaccine. Our results also confirmed that the mice inoculated with the VSV-based recombinant viruses were safe and induced a robust immune response.

The recombinant viruses had similar growth characteristics to VSV-rwt, and the insertion of antigen genes into the VSV backbone did not affect the morphology of VSV (He et al., 2023). In the present study, the results indicated that VSV-p35 and VSV-p72 exhibited stable transmission for up to 20 generations in BHK-21 cells, with robust maintenance of gene inheritance and efficient expression of exogenous proteins. The pathogenicity study demonstrated that none of the BALB/c mice immunized with the recombinant viruses had any adverse effects, including undetectable body weight loss and mortality. The recombinant viruses exhibited transient infection in blood and tissues. The BALB/c mice immunized with the recombinant viruses were normal with no obvious tissue lesions. Analysis of a routine blood examination revealed that recombinant viruses had activated the immune system of mice, as evidenced by a reduction in total white blood cell count during early vaccination with the recombinant viruses. Monocyte and neutrophil cell counts were decreased due to their participation in phagocytosis of the virus. Subsequently, exogenous antigens activated the host immune response and resulted in an increase in lymphocyte count, which is consistent with the regular pattern of virus infection. These results suggest that recombinant virus as a candidate for a live attenuated vaccine prototype was safe for BALB/c mice. The findings are in line with a previous study which showed that a replication-competent recombinant VSV vaccine expressing the EBOV glycoprotein was safe and efficacious in nonhuman primates (Jones et al., 2005).

The immunogenicity of vaccine candidates for ASFV has been tested, but only either a humoral or a cellular immune response was elicited. Previous studies have demonstrated that vaccination with recombinant p30 and p54 or a fusion protein p54/30 produced neutralizing antibodies in pigs, but the cellular immune response was suboptimal, resulting in an unsatisfactory protective effect (Lacasta et al., 2014). In the present study, a single dose of VSV-p35, VSV-p72, and VSV-p35 + p72 was sufficient to elicit high levels of specific antibodies for about 42 days, and the expression of IL-2, IFN-γ, and TNF-α in the spleen lymphocytes of immunized mice was increased. These findings were further supported by Kennedy et al.’s report, which confirmed that rVSVΔG-ZEBOV-GP induced lasting protection and sustained immunity (Kennedy et al., 2017). Furthermore, the depletion of CD8^+^ T lymphocytes in pigs immunized with ASFV can disrupt protective immunity against virulent strains, and cellular immunity is crucial in the development of effective vaccines against ASFV (Oura et al., 2005; Takamatsu et al., 2013). The findings demonstrate that mice vaccinated with recombinant viruses elicited robust antigen-specific T cell responses, characterized by significantly elevated levels of CD8^+^T and IFN-γ^+^ compared to the control groups. Taken together, we demonstrated that the recombinant viruses in mice were safe and triggered robust immune responses, which suggests that VSV as an antigen delivery platform is a safe and reliable option. There were limitations in conducting a live ASFV challenge experiment due to restricted experimental conditions. Therefore, it is necessary to evaluate the protective efficacy of the vaccine prototype in pigs in future experiments.

## 6 Conclusion

In summary, this is the inaugural report utilizing VSV strains as a vaccine vector for expressing ASFV antigen genes. The recombinant viruses replicated efficiently in BHK-21 cells and were shown to be safe in BALB/c mice. Moreover, a single dose of the vaccine prototype induced and sustained high-level specific humoral and cellular immune responses, suggesting that VSV may be a potential and effective antigen delivery vector.

## Data availability statement

The original contributions presented in the study are included in the article/Supplementary material, further inquiries can be directed to the corresponding author.

## Ethics statement

The animal study was approved by the Animal Care and Use Committee of Lanzhou Veterinary Research Institute (LVRI), Chinese Academy of Agricultural Sciences. The study was conducted in accordance with the local legislation and institutional requirements.

## Author contributions

YM: Conceptualization, Data curation, Investigation, Visualization, Writing – original draft. JS: Formal analysis, Writing – review & editing. WL: Formal analysis, Writing – review & editing. SG: Formal analysis, Writing – review & editing. DP: Methodology, Supervision, Writing – review & editing. CM: Methodology, Supervision, Writing – review & editing. SY: Methodology, Supervision, Writing – review & editing. ZH: Methodology, Supervision, Writing – review & editing. GZ: Methodology, Supervision, Writing – review & editing. XQ: Funding acquisition, Project administration, Writing – review & editing. HC: Funding acquisition, Project administration, Writing – review & editing.
