# Corrigendum: A vesicular stomatitis virus-based African swine fever vaccine prototype effectively induced robust immune responses in mice following a single-dose immunization

**DOI:** 10.3389/fmicb.2025.1585665

**Published:** 2025-03-18

**Authors:** Yunyun Ma, Junjun Shao, Wei Liu, Shandian Gao, Decai Peng, Chun Miao, Sicheng Yang, Zhuo Hou, Guangqing Zhou, Xuefeng Qi, Huiyun Chang

**Affiliations:** ^1^State Key Laboratory for Animal Disease Control and Prevention, Lanzhou Veterinary Research Institute, Chinese Academy of Agricultural Sciences, Lanzhou, Gansu, China; ^2^College of Veterinary Medicine Northwest A&F University, Yangling, Shanxi, China

**Keywords:** African swine fever virus, vaccine prototypes, vesicular stomatitis virus, safety, immune potency

In the published article, there was an error regarding the affiliation(s) for [Yunyun Ma]. As well as having affiliation(s) [1], they should also have [College of Veterinary Medicine Northwest A&F University, Yangling, Shanxi, China].

The authors apologize for this error and state that this does not change the scientific conclusions of the article in any way. The original article has been updated.

