# Supplementary material for: A vesicular stomatitis virus-based African swine fever vaccine prototype effectively induced robust immune responses in mice following a single-dose immunization
Source: Front Microbiol. 2024 Jan 5;14:1310333. doi: 10.3389/fmicb.2023.1310333 (PMC10797088; doi:10.3389/fmicb.2023.1310333)
Supplement: Supplementary file 1 [file Table_1.DOCX]

**TABLE 1 The immunization protocol of the recombinant virus live vector vaccines in BALB/c mice.**

| **Group** | **Doses (TCID_50_/mouse)** | **Number of animals** | **Inoculated route** |
| --- | --- | --- | --- |
| VSV-p72 | 5 × 10^6^ | 8 | i.m |
|  | 2.5 × 10^6^ | 8 | i.m |
|  | 1 × 10^6^ | 8 | i.m |
|  | 2.5 × 10^5^ | 8 | i.m |
|  | 1 × 10^5^ | 8 | i.m |
| VSV-p35 | 5 × 10^6^ | 8 | i.m |
|  | 2.5 × 10^6^ | 8 | i.m |
|  | 1 × 10^6^ | 8 | i.m |
|  | 2.5 × 10^5^ | 8 | i.m |
|  | 1 × 10^5^ | 8 | i.m |
| VSV-p35+p72 | 5 × 10^6^ | 8 | i.m |
|  | 2.5 × 10^6^ | 8 | i.m |
|  | 1 × 10^6^ | 8 | i.m |
|  | 2.5 × 10^5^ | 8 | i.m |
|  | 1 × 10^5^ | 8 | i.m |
| VSV-rwt | 5 × 10^6^ | 8 | i.m |
|  | 2.5 × 10^6^ | 8 | i.m |
|  | 1 × 10^6^ | 8 | i.m |
|  | 2.5 × 10^5^ | 8 | i.m |
|  | 1 × 10^5^ | 8 | i.m |
| PBS | — | 40 (Total) | i.m |
